# Positive attitudes to advance care planning – a Norwegian general population survey

**DOI:** 10.1186/s12913-021-06773-x

**Published:** 2021-08-02

**Authors:** Trygve Johannes L. Sævareid, Reidar Pedersen, Morten Magelssen

**Affiliations:** Centre for Medical Ethics, University of Oslo, Kirkeveien 166, Frederik Holsts hus, 0450 Oslo, Norway

**Keywords:** Advance care planning, Attitudes, General population, Public health care

## Abstract

**Background:**

Authorities recommend advance care planning and public acceptance of it is a prerequisite for widespread implementation. Therefore, we did the first study of the Norwegian public with an aim of getting knowledge on their attitudes to issues related to advance care planning.

**Methods:**

An electronic survey to a nationally representative web panel of Norwegian adults.

**Results:**

From 1035 complete responses (response rate 40.7%), we found that more than nine out of ten of the general public wanted to participate in advance care planning, believed it to be useful for many, and wanted to make important healthcare decisions themselves. Almost nine out of ten wanted to be accompanied by next of kin during advance care planning. Most (69%) wanted health care personnel to initiate advance care planning and preferred it to be timed to serious illness with limited lifetime (68%). Only about 9% stated that health care personnel should have the final say in healthcare decisions in serious illness.

**Conclusions:**

Developing and implementing advance care planning as a public health initiative seems warranted based on the results of this study. Patient perspectives should be promoted in decision-making processes. Nevertheless, training of health care personnel should emphasise voluntariness and an individual approach to initiating, timing and conducting advance care planning because of individual variations.

## Background

Advance care planning (ACP) may prevent decision-making conflicts by involving patients, their next of kin and health care personnel in discussions relevant for decision making before decisions are needed [1]. This is pertinent because end-of-life decision-making is often characterized by ethical problems that can give rise to moral distress for health care personnel [2]. Other potential benefits of ACP implementation range from increasing health care personnel’s comfort in engaging in end-of-life communication [3] to making treatment at the end of life more concordant with patient preferences [4]. ACP is thus recommended by authorities [5] and the European Association for Palliative Care [6].

Importantly, although policy makers endorse ACP and health care personnel recognize it, ACP has little value if patients and the public do not want it. Public opinion may help policymakers develop and implement targeted health care interventions [7] that the public want. While ACP in Norway is seldom (yet increasingly) used [8], it is recommended by the authorities [9]. Meanwhile, in the Norwegian legal framework, ACP is inadequately addressed. Certainly, Norwegian law acknowledges a patient’s right to consent to care and to participate in decision-making. However, Norwegian health laws can be considered quite paternalistic with much power given to physicians and other health care personnel, especially during emergency situations (health personnel act §7) and when patients have reduced/lost competence to consent (patient and user rights act §4–6) [10]. For patients without decision-making competency, the role of next of kin is limited to relaying what the patient has previously expressed about his/her wishes, and does not involve making decisions on the patient’s behalf (patient and user rights act). Information about the patient’s prior wishes shall be obtained by health care personnel and considered when making decisions. Therefore, for these patients to be involved in decision-making processes, it is pressing to talk with them while they are still able. ACP can facilitate this, but Norwegian public opinion about ACP is unknown.

Internationally, there are studies on older people’s attitudes towards ACP [11, 12] and on the public’s attitudes [13–16]. They indicate that while most people have thought about end-of-life care and decision-making [14] there is a wish for information about prognosis, diagnosis and available treatments when facing life-limiting illness [13, 15]. Furthermore, most seem willing to participate in conversations on end-of-life care [11, 12, 16]. We hypothesized that similar attitudes to ACP prevail in the Norwegian public. If so, it could give the national authorities an incentive to and a warrant for doing more in implementing ACP as a public health initiative. Consequently, we aimed to get knowledge on the Norwegian public’s attitudes on involvement in and preferences for decision-making processes, the usefulness of and willingness to participate in ACP, involvement of next of kin, initiation and timing of ACP, and study demographic determinants of ACP attitudes.

## Methods

### Design and population

A cross-sectional survey study was conducted. In December 2019, an electronic questionnaire was distributed by the commercial firm Kantar to members of their nationally representative web panel of adults [17], via email. The web panel has 40,000 members who have agreed to answer surveys. As compensation, panel members receive points, which can be spent on gifts, gift cards or donated to charities. Number of points received varies depending on the length of the survey, and panelists are informed about the number of points they will receive when invited to do a survey. Panel members were invited successively until the target number of 1000 responses had been reached. Reporting adheres to the STROBE checklist [18].

### Questionnaire

The questionnaire had three sections; this study regards the final section, which was about ACP, while the first two sections explored issues on priority settings in health care and end-of-life decision-making. The questionnaire was in Norwegian, and was designed based on international research on ACP [11, 12], policies, philosophical traditions on autonomy [19], and supported decision-making [20]. It was developed through discussions among the authors. Laypersons pilot tested the electronic version in two stages. Survey questions with corresponding response alternatives are shown in Table 1.

**Table. 1:** Survey questions and response alternatives

Survey questions	Response alternatives
How much do you agree or disagree that “In general it is important for me to make important decisions about healthcare myself”?	Fully disagree
	Disagree somewhat
	Neither nor
	Agree somewhat
	Fully agree
If you were to become seriously ill, who do you think should have the final say in healthcare decisions?	Myself
	Next of kin
	Health care personnel
I think advance care planning can be useful for many patients?	Fully disagree
	Disagree somewhat
	Neither nor
	Agree somewhat
	Fully agree
If given an opportunity, I would like to participate in advance care planning	Fully disagree
	Disagree somewhat
	Neither nor
	Agree somewhat
	Fully agree
If I were to participate in advance care planning I would want a next of kin with me	Fully disagree
	Disagree somewhat
	Neither nor
	Agree somewhat
	Fully agree
Who should ideally initiate advance care planning?	Myself
	Next of kin
	General practitioner
	Hospital physician
	Nursing home staff
	Other health care personnel
	Preferably nobody
When should advance care planning ideally take place?	The sooner the better
	By 60 years
	By 70 years
	By 80 years
	At the time of serious/chronic illness and limited lifetime, independent of age
	Preferably never

In the questionnaire, ACP was defined and described as “a conversation between patient, health care professional and often also relatives. It is about what is important to the patient, as well as preferences for medical treatment and care. Advance care planning is often offered to elderly patients and patients with chronic and serious illnesses. Through such conversations, it becomes easier to act in accordance with the patient’s wishes and values at a later date, especially if the patient is no longer able to choose for themselves.” The definition and description was inspired by other definitions [21, 22] and our own and others’ research on ACP [3, 23–26].

### Statistical analyses

Statistical analyses were performed with IBM SPSS Statistics version 26. Responses were weighted according to gender, age, and geographical region so that responses from groups underrepresented among the respondents were given increased weight (Table 2). Analyses were performed on weighted data.

**Table. 2:** Respondent characteristics. *N* = 1035

	Unweighted (N (%))	Weighted (N (%))
Age		
Mean	53.5 years	47.8 years
Under 30	125 (12.1)	210 (20.3)
30–44	188 (18.2)	266 (25.7)
45–59	291 (28.1)	266 (25.7)
60+	431 (41.6)	293 (28.3)
Female	534 (51.6)	514 (49.6)
Highest completed education		
Primary school	48 (4.6)	39 (3.8)
Upper secondary school	269 (26)	296 (28.6)
Higher education/vocational school up to 4 years	464 (44.9)	445 (43)
Higher education more than 4 years	254 (24.5)	255 (24.6)
Religious view of life		
Christian	533 (51.5)	485 (46.8)
Muslim	6 (0.6)	9 (0.9)
Other religion	13 (1.3)	16 (1.5)
Non-religious	420 (40.6)	462 (44.7)
Do not wish to state or NA	63 (6)	63 (6.1)

We performed multiple logistic regression analyses to calculate odds ratios with 95% confidence intervals for the relationships between decision-making preferences and ACP attitudes (dependent variables), and demographic characteristics (independent variables). Dependent variables were scored on a five-point Likert scale with “fully disagree” (=1) and “fully agree” (=5) as scale anchors.

We dichotomized independent and dependent variables. The dependent variables were dichotomized into “Disagree/neither nor” [1–3] and “Agree” [4, 5]. Dichotomized independent variables in the analysis included: religious view of life (“non-religious” indicated “atheist/agnostic” and “non-religious”), highest completed education (“higher education” indicated college/university degree of a length of 3 or more years), and trust in the public health care services (“high trust” indicated response ranging from 7 to 10 on a scale from “no trust” (=1) to “full trust” (=10)). In addition, age, gender and contact with general practitioner (GP) were independent variables. Dichotomized variables excluded ‘do not wish to state’ responses, which led to missing cases in the analysis.

## Results

2540 panel members were invited, and 1076 responded. We received 1035 complete responses (response rate 40.7%). Information on the demographic characteristics of the respondents is presented in Table 2.

### ACP attitudes and decision-making preferences

A majority of respondents would like to participate in ACP (91.8%) and even more thought ACP could be useful for many patients (93.9%). Many respondents agreed that it was important for them to make important healthcare decisions themselves (92.5%). Interestingly, only 9.2% of respondents wanted health care personnel to have the final say in healthcare decisions, which was fewer than the alternatives ‘myself’ or ‘next of kin’. However, a majority wanted health care personnel to initiate ACP (69%), with most preferring the GP to initiate ACP (33%). Although many were positive to ACP, most seemed to be most inclined to participate at a time of chronic/serious illness and limited lifetime (68.6%; Tables 3 and 4).

**Table. 3:** Attitudes on decision-making, usefulness of and involvement in advance care planning

Respondents *N* = 1035^a^ (%)					
Survey question	Fully disagree	Disagree somewhat	Neither nor	Agree somewhat	Fully agree
Generally, for me it is important to make important healthcare decisions myself	10 (0.9)	17 (1.7)	49 (4.7)	261 (25.2)	675 (67.2)
I think advance care planning can be advantageous for many patients	6 (0.6)	8 (0.8)	31 (3.0)	166 (16.1)	806 (77.9)
I would like to participate in advance care planning	3 (0.3)	13 (1.2)	38 (3.7)	136 (13.1)	814 (78.7)
I would want a next of kin with me, if I was to participate in advance care planning	10 (1.0)	21 (2.1)	83 (8.0)	182 (17.6)	717 (69.2)

^a^The highest number of missing cases was 31 for any of these variables, which are ‘do not wish to state’ or ‘Not applicable’ (NA) responses

**Table. 4:** Attitudes on final say in decisions, initiation and timing of advance care planning

Survey question	Response alternatives	N = 1035^a^ (%)
If you get seriously ill, who would you like to have the final say in healthcare decisions?	Myself	723 (69.9)
	Next of kin	171 (16.5)
	Health care personnel	96 (9.2)
Who should initiate advance care planning?	Myself	193 (18.6)
	Next of kin	62 (5.9)
	General practitioner	345 (33.4)
	Hospital physician	275 (26.6)
	Nursing home staff	78 (7.5)
	Other health care personnel	16 (1.5)
	Preferably nobody	11 (1)
Preferred timing of advance care planning	The sooner the better	170 (16.4)
	By 60 years	39 (3.8)
	By 70 years	42 (4)
	By 80 years	15 (1.5)
	At the time of serious/chronic illness and limited lifetime, independent of age	710 (68.6)
	Preferably never	11 (1)

^a^‘Do not wish to state’ or ‘NA’ responses varied from 45 to 56 for these variables

### Factors associated with decision-making preferences and ACP attitudes

The proportion who wanted to participate in ACP was higher for female respondents and for those with a “high trust”; these were statistically significant associations (Table 5). Persons 60 years or older and “non-religious” tended to be more likely to want to participate in ACP, although not statistically significant. Females were (statistically significant) more likely to: want to make important healthcare decisions themselves, think that ACP can be useful, and want to be accompanied by their next of kin during ACP. Persons with “high trust” were more likely to want to make important healthcare decisions themselves and think that ACP can be useful (statistically significant) and want to be accompanied by their next of kin during ACP (approximating statistical significance). In addition, the association between being “non-religious” and thinking ACP can be useful was statistically significant. We found no significant association between attitudes toward ACP and number of contact with GP, income and education.

**Table 5:** Characteristics of the general population associated with advance care planning attitude^a^

Characteristics	Advance care planning attitudes			
	I want to make important healthcare decisions myself^b^ OR^c^ (95% CI^d^)	Advance care planning can be useful for many	I would like to participate	I would want a next of kin with me
2 or more contacts with GP in the past 12 months	1.40 (0.85 – 2.30)	0.88 (0.38 – 2.0)	0.9 (0.45 – 1.8)	1.40 (0.89 – 2.22)
Female	1.78^e^ (1.00 – 3.15)	3.61^e^ (1.41 – 9.26)	7.32^e^ (2.74 – 19.57)	3.93^e^ (2.30 – 6.72)
Age ≥ 60 years	0.62 (0.35 – 1.09)	1.09 (0.45 – 2.68)	2.16 (0.87 – 5.38)	1.14 (0.66 – 1.95)
Non-religious	1.15 (0.67 – 1.98)	2.37^e^ (1.01 – 5.54)	1.7 (0.85 – 3.39)	0.74 (0.47 – 1.18)
Higher education	1.26 (0.72 – 2.22)	1.94 (0.88 – 4.28)	1.54 (0.78 – 3.05)	1.10 (0.68 – 1.77)
High trust in the public health care services	2.04^e^ (1.17 – 3.54)	2.22^e^ (1.01 – 4.90)	1.98^e^ (1.00 – 3.92)	1.60 (0.98 – 2.60)

^a^Odds Ratio with 95% confidence interval from multiple logistic regression analyses

^b^Missing cases varied between 20.2 – 21.3% for these analyses

^c^Odds Ratio

^d^Confidence interval

^e^indicates a statistically significant result

## Discussion

This study indicates that more than nine out of ten in the general Norwegian population want to take part in ACP, want to make important healthcare decisions themselves and believe ACP can be useful for many. Many would participate together with their next of kin. Even though most thought that ACP should occur at the time of serious/chronic illness and limited lifetime and health care personnel should initiate ACP, only a minority thought health care personnel should have the final say in health care decisions. Females and respondents with “high trust” in the public health care services were more likely to be positive about both making important healthcare decisions themselves, viewing ACP beneficial and wanting to participate in ACP.

### Public attitudes on ACP participation

Among barriers to doing ACP is a conception among health care personnel that patients do not want to participate in such discussions [27, 28]. Our results indicate that this is a misconception. The general public are mostly willing to participate in ACP according to other surveys in the Netherlands [14], South Korea [16] and in Singapore [29], although our results indicate even higher willingness in Norway. In addition, most respondents in a community intervention study were comfortable discussing their own end-of-life care [30]. In a systematic review, older persons have been found to be willing to discuss their end-of-life care [11]. Some older persons placed low value on ACP because they were comfortable having others decide for them [25]. However, ACP has been well received by patients, next of kin and health care personnel [3].

The overall willingness to and comfort in discussing end-of-life matters should make ACP an intriguing public health initiative – partly because ACP has a potential for contributing to improving end-of-life care [4, 31]. Knowledge of the public’s opinion is prudent when developing health care initiatives and for allocation of scarce resources. If the public is positive to initiatives, such as ACP, authorities and health enterprises may be more inclined to support their implementation. However, ACP is a complex intervention [32, 33], which makes successful implementation more difficult. The complexity of ACP coupled with a lack of implementation support [34], plus lack of public knowledge may contribute to explaining its low uptake [11, 14, 35]. In addition, overcoming barriers is important in achieving widespread use of ACP. Among reported barriers are uncertainty, lack of comfort and lack of knowledge among health care personnel in doing ACP [13, 28, 36–38]. Implementation of ACP thus warrants proper education, training and follow-up of health care personnel and leaders.

Patient participation in ACP improves when next of kin participate together with the patient [37, 39]. Furthermore, next of kin may be valuable in supporting patients during ACP, particularly for patients with reduced decision-making capacity. Next of kin support may strengthen the decision-making capacity of patients and is recommended as part of supported decision-making [20]. If the patient is willing, next of kin should be invited to ACP discussions.

### Initiation and timing of ACP

The majority view in this study was that health care personnel should initiate ACP, which supports others findings [14, 25, 40]. Health care personnel must therefore recognize their responsibility in initiating the discussions in order to stimulate higher ACP uptake. However, initiation of ACP may be hampered by uncertainty about when such discussions are timely [25, 41].

Most respondents in this study preferred to participate in ACP at a time of chronic/serious illness and limited lifetime, which was higher compared to others [42]. However, that might run contrary to the idea of ACP being held while persons are still able to communicate [22]. In addition, health care personnel have indicated that ACP close to dying is “too close and tense” and a peaceful atmosphere is needed [43]. Health care personnel may worry about causing patients harm by having untimely ACP discussions [44] and this can increase the uncertainties about when ACP is timely. There is little indication that ACP causes distress to patients [25, 45], although it can be experienced as unpleasant and difficult issues should be addressed without “going too far” [39]. In sum, health care personnel need not be overly worried about ACP harming patients. Instead, a patient-centered approach to training, implementation and practice of ACP should be pursued. Identifying an optimal timing of ACP is difficult, because preferred timing is individual [25]. Consequently, optimal timing of ACP is when the patient is ready [39].

ACP has developed from advance directives (living wills) into the communicative process it is today during the past few decades [21, 46]. In Norway, and likely several other countries, ACP has been a tool available for health care services only within the last decade. Most patients accept ACP [47], few experience ACP as distressing [25] and Bhavsar and colleagues declared the “death of outrage over talking about dying” [48]. However, as a public health initiative, it is essential to keep in mind that not all patients want to participate in ACP [11]. Importantly, ACP participation is voluntary. This should be communicated in invitations to participate in order to give patients an opportunity to decline participation. An informative invitation to ACP that recognizes the patient’s right to decline may ease initiation and timing burdens for health care personnel.

### Attitudes on decision-making

Fewer than one out of ten respondents wanted health care personnel to have the final say in healthcare decisions, if they get seriously ill. This is another striking result from this study. The survey question did not state that the patient lacked decision-making competence and competent Norwegian patients do have rights in consenting to healthcare decisions. Nevertheless, Norwegian law give physicians vast judicial power in decision-making when patients lack decision-making competence or in emergency care situations (independent of decision-making competence) [10]. However, our results may indicate that the public values patient, next of kin or shared decision-making over leaving decisions to health care personnel.

Respondents wanting next of kin rather than health care personnel to have the final say is supported by other studies [10, 12, 49]. In the Norwegian legal context, opinions of next of kin should not be decisive in end-of-life decision-making. Next of kin should instead contribute by relaying previous statements and values stated by the patient. Contrary to Norwegian law, there are indications that a majority of the Norwegian populace want decision-making authority [10]. Although in practice, next of kin seems to be receiving more responsibility in end-of-life decision-making than they ought to according to the law [50]. In addition, getting end-of-life decision-making responsibilities can be experienced burdensome for next of kin [51, 52].

### Factors associated with decision-making preferences and ACP attitudes

Here we discuss factors associated with decision-making preferences and ACP attitudes. Most of the associations we found were in line with others research. Women have been found more likely to discuss ACP with family and friends and to engage in more ACP practices [53] and to think about end-of-life decision-making [14]. Older persons are more likely to being willing to engage in an ACP discussion [29], more likely to being engaged in ACP activities [53] and having thought about end-of-life decision-making [14]. However, contrary to our results, a survey among the Dutch public found that little trust in physicians complying with their wishes about medical care/treatment in the last days of life indicated a preference for making their own decisions [14].

Non-religious persons seem more engaged in ACP [14]. Adding to this is the higher likelihood of “non-religious” persons to refuse life-sustaining treatment at end of life [54]. A possible reason for religious persons more often opting to not participate in ACP and being more likely to accept life-sustaining treatment may be a desire not to interfere with God’s plan [55]. Furthermore, religious persons may be more inclined to view life as sacred – making future planning or life-sustaining treatment less attractive – or finding comfort in being in the care of God.

Differences in ACP attitudes between different sociodemographic groups give knowledge for policy makers and clinicians on developing and targeting ACP interventions. Importantly though, such variations emphasize the importance of a principle relevant to ACP involvement and end-of-life decision-making – voluntariness. If participation in ACP is not voluntary, health care services risk losing the public’s trust and compromising patient beneficence.

### Strengths and limitations

This study is population-based founded on a representative sample of the Norwegian population. The moderate response rate might conceivably have contributed to a non-response bias.

As mentioned in the Methods section, the questionnaire consisted of three sections – priority settings, end-of-life decision-making, and ACP. We do not view ACP as end-of-life decision-making, rather a tool to prepare for this end-of-life decision-making in a way that promotes patient autonomy also if the patient is no longer competent to consent. However, the ACP section succeeding end-of-life decision-making may have prompted some respondents to think of ACP as end-of-life decision-making.

Our description of ACP included that “Advance care planning is often offered to elderly patients and patients with chronic and serious illnesses”. When asked about preferred timing of ACP, responses may have been influenced by similarities in the formulation of the most preferred response alternative (“At the time of serious/chronic illness and limited lifetime, independent of age”) and the information we provided on when ACP is usually offered.

ACP is today considered a process consisting of several conversations [21, 22]. Our definition did not sufficiently recognize ACP as a process. A more precise definition might have affected responses. However, we believe this would have led to more responses that are positive rather than negative, because viewing ACP as a process might offset any worries among respondents’ that ACP would not recognize changes to their needs and wishes.

## Conclusions

ACP was largely wanted and viewed as useful for many patients, according to this survey of Norwegian adults. Developing and implementing ACP as a public health initiative thus seems warranted. The public responses further implicated that health care services should focus on promoting patient autonomy in decision-making processes. Health care services may want to tailor information about ACP and recruitment strategies to certain groups of society. For instance, men were less likely to want to participate in ACP compared to women in this study and developing ACP to the needs of men might increase the acceptance of ACP among them. Nevertheless, training of health care personnel should emphasize voluntariness and an individual approach to initiating, timing and conducting ACP. That would safeguard variations among patients’ preferences for involvement and could strengthen public trust in the health care services.

## Data Availability

The datasets used and/or analyzed during the current study are available from the corresponding author on reasonable request.

## Abbreviations

ACP: Advance care planning
GP: General practitioner
